# Characterization of the oxygen-tolerant formate dehydrogenase from *Clostridium carboxidivorans*

**DOI:** 10.3389/fmicb.2024.1527626

**Published:** 2025-01-13

**Authors:** Eva-Maria Brouwer, Hitesh K. R. Medipally, Saskia Schwab, Shanshan Song, Marc M. Nowaczyk, Martin Hagemann

**Affiliations:** ^1^Department of Plant Physiology, Institute of Biosciences, University of Rostock, Rostock, Germany; ^2^Department of Plant Biochemistry, Faculty of Biology and Biotechnology, Ruhr-University Bochum, Bochum, Germany; ^3^Science for Life Laboratory, School of Engineering Sciences in Chemistry, Biotechnology and Health, KTH—Royal Institute of Technology, Stockholm, Sweden; ^4^Department of Biochemistry, Institute of Biosciences, University of Rostock, Rostock, Germany; ^5^Department of Life, Light and Matter, Interdisciplinary Faculty, University of Rostock, Rostock, Germany

**Keywords:** enzyme, photosynthesis, redox, site-specific mutants, carbon fixation

## Abstract

Fixation of CO_2_ into the organic compound formate by formate dehydrogenases (FDHs) is regarded as the oldest autotrophic process on Earth. It has been proposed that an FDH-dependent CO_2_ fixation module could support CO_2_ assimilation even in photoautotrophic organisms. In the present study, we characterized FDH from *Clostridium carboxidivorans* (*cc*FDH) due to its ability to reduce CO_2_ under aerobic conditions. During the production of recombinant *cc*FDH, in which the selenocysteine codon was replaced by Cys, we were able to replace the W with Mo as the transition metal in the *cc*FDH metal cofactor, resulting in a two-fold increase of 6 μmol formate min^−1^ in enzyme activity. Then, we generated *cc*FDH variants in which the strict NADH preference of the enzyme was changed to NADPH, as this reducing agent is produced in high amounts during the photosynthetic light process. Finally, we showed that the native *cc*FDH can also directly use ferredoxin as a reducing agent, which is produced by the photosynthetic light reactions at photosystem I. These data collectively suggest that *cc*FDH and, particularly, its optimized variants can be regarded as suitable enzymes to couple formate production to photosynthesis in photoautotroph organisms, which could potentially support CO_2_ assimilation via the Calvin–Benson–Bassham (CBB) cycle and minimize CO_2_ losses due to photorespiration.

## Introduction

Formate is one of the simplest organic compounds and serves as a crucial source of carbon and reducing power for living cells. It can be produced through various routes, including the electrochemical reduction of CO_2_, photoreduction of CO_2_, hydrogenation of CO_2_, selective oxidation of biomass, partial oxidation of natural gas, and hydration of syngas (i.e., a mixture of hydrogen and carbon monoxide) (Bar-Even, 2016). Formate dehydrogenases (FDHs, EC1.17.1.9) are a heterogeneous group of proteins found in eukaryotes and prokaryotes (Jormakka et al., 2003; Yu et al., 2014), with some of them having the ability to catalyze the reversible reduction of CO_2_ to formate. In nature, many anaerobic bacteria and archaea can efficiently fix CO_2_ (or CO) into formate via the Wood–Ljungdahl pathway (Ragsdale and Pierce, 2008), which is the oldest autotrophic CO_2_ assimilation route on Earth (Moody et al., 2024). In recent years, the increased release of CO_2_ into the atmosphere due to human activities, mostly from burning fossil carbon sources for energy production, has been identified as the main driver of climate change (Lee et al., 2023). To mitigate this impact, carbon-neutral processes using FDHs to convert CO_2_ into organic compounds have become a focus of biotechnology and synthetic biology (Alissandratos et al., 2013a; Bar-Even, 2016).

FDHs can be divided into two major classes, based on their metal content and the subsequent chemical strategy employed by the active site to carry out formate oxidation (Maia et al., 2017). One class, metal-center-independent FDH proteins, belongs to the superfamily of D-specific dehydrogenases of 2-hydroxy acids (Tishkov and Popov, 2006; Maia et al., 2017). FDHs from this class usually catalyze formate oxidation coupled with the reduction of NAD^+^ to NADH. The other class, metal-center-dependent FDH proteins, contains a complex inventory of redox-active centers and is usually sensitive to oxygen (Maia et al., 2015). CO_2_-fixing FDHs in anaerobic bacteria and archaea performing the Wood–Ljungdahl pathway belong to the class of metal-center-dependent enzymes. These proteins contain molybdenum, tungsten, or other transition metals in their active site, coordinated by pyranopterin cofactors, to facilitate formate degradation or CO_2_ reduction. Additionally, to promote electron transport, they usually contain several iron–sulfur clusters. This group of FDHs is of special interest as they provide enzymes that could catalyze efficient CO_2_ fixation when coupled with naturally occurring formate assimilation. In the case of the Wood–Ljungdahl pathway, formate assimilation occurs via a formate tetrahydrofolate ligase that produces the C1 component formyl-tetrahydrofolate (formyl-THF). Formyl-THF is found ubiquitously in all living organisms within C1 metabolism, and thus, in terms of synthetic biology, it represents a potential entry point to various metabolic networks.

Recently, the establishment of FDH-mediated CO_2_ reduction into formate in photoautotrophic organisms was proposed to support CO_2_ fixation via the Calvin–Benson–Bassham (CBB) cycle (Bar-Even, 2018). In all organisms that perform oxygenic photosynthesis—cyanobacteria, algae, and plants—photosynthetic CO_2_ fixation is catalyzed by the key enzyme ribulose 1,5-bisphosphate carboxylase/oxygenase (RubisCO). However, RubisCO has been identified as a limiting factor for efficient CO_2_ fixation under current atmospheric conditions because the enzyme is rather slow and has a low CO_2_ affinity. To offset these limitations, organisms rely on high amounts of RubisCO. The CO_2_ affinity of RubisCO enzymes varies between different organisms. For example, it is approximately 10 times lower in land plants than in most cyanobacteria and algae, which is compensated for by the evolution of a CO_2_-concentrating mechanism in the latter photoautotrophs (For a review, see Badger and Sharwood, 2023). Furthermore, RubisCO shows the oxygenase side reaction, where oxygen (O_2_) is fixed instead of CO_2_, leading to the formation of the toxic intermediate 2-phosphoglycolate, which needs to be salvaged into CBB intermediates at the expense of CO_2_ and energy losses via photorespiration (Tcherkez et al., 2006; Bar-Even, 2018; Kern et al., 2020). The salvaging of 2-phosphoglycolate in the photorespiratory cycle includes steps of the C1 metabolism. Hence, in photoautotrophs, the formyl-THF resulting from formate assimilation can be introduced into plant metabolism via photorespiration, after stepwise reduction to methylene-THF, which, together with glycine, serves for serine biosynthesis at serine hydroxymethyltransferase (SHMT). It has been discussed that an increased pool of methylene-THF due to efficient formate assimilation could turn photorespiration into less CO_2_-releasing or even CO_2_-fixing when the glycine-decarboxylase reaction is reversed. However, this has been only observed in synthetic biology studies involving *Escherichia coli* (e.g., Yishai et al., 2018) and not in photoautotrophs. Recently, the formate-assimilation pathway, including a reversed glycine decarboxylase flux, was successively established in *E. coli*, proving the previously designed CO_2_-fixing shunt as kinetically feasible and functional (Yishai et al., 2017; Bang and Lee, 2018; Döring et al., 2018; Kim et al., 2020). These synthetic biology approaches confirm that artificial formate assimilation pathways are compatible with cellular metabolism, but they have not yet seen widespread practical application.

To establish CO_2_ reduction into formate in oxygenic phototrophs, a suitable FDH is needed, one that not only efficiently reduces CO_2_ but is also oxygen-tolerant and can use NADPH or ferredoxin as the electron source. A promising candidate for expression in phototrophs is the NAD-dependent FDH from acetogenic *Clostridium carboxidivorans*, *cc*FDH. It has been reported to preferentially reduce CO_2_ to formate even in the presence of O_2_ (Alissandratos et al., 2013b). In the present study, we aimed to biochemically characterize this enzyme and take the first steps in optimizing its acceptor capabilities for future application in oxygenic phototrophs.

## Materials and methods

### Gene synthesis and cloning

The nucleotide sequence of the *cc*FDH (Acc. No. UniProt E2IQB0) was synthesized by Baseclear (Leiden, Netherlands), in which codon 139 for selenocysteine (Sec) was changed to TGC encoding cysteine (139Cys; see Supplementary Figure S1). The gene was inserted via *Nhe*I/*Bgl*II into pET28a (Novagen) for subsequent expression with an N-terminal His_6_-tag. The resulting construct was confirmed by sequencing (Microsynth, Göttingen, Germany). Further amino acid substitutions were introduced by site-directed mutagenesis using primers listed in Supplementary Table S1.

### Bioinformatic analysis

The homology model of the *cc*FDH was created using the SWISS-MODEL (Waterhouse et al., 2018; Studer et al., 2020) using FDH-H from *E. coli* as the template (64.01% sequence identity; QMEANDisCo Global: 0.83 ± 0.05). The conservation of amino acids was depicted using the ConSurf web server (Ashkenazy et al., 2016).

### Protein expression and purification

Aerobic protein expression was performed in the strain *E. coli* BL21(DE3) using LB medium (Roth, Germany). Pre-cultures were diluted to an OD_600_ of 0.1 and incubated until they reached 0.6–0.8. The expression was induced by the addition of 1 mM IPTG. The cells were shaken at room temperature (RT) for 4 h. For co-factor determination, the *E. coli* cells were grown in M9 minimal medium either without any additions, with 1 mM Na_2_MoO_4_, or with 1 mM Na_2_WO_4_. For all other experiments, LB was supplemented with 1 mM Na_2_MoO_4_.

The cells were homogenized in a lysis buffer [50 mM HEPES pH 7.8, 100 mM NaCl] after incubation with lysozyme at RT, followed by sonication on ice. The lysate was cleared by centrifugation (30,000 × g, 30 min, 4°C), and the protein was immobilized via IMAC, washed two times with 10 batch volumes of a wash buffer (50 mM HEPES pH 7.8, 100 mM NaCl, 50 mM imidazole). Elution was performed with two batch volumes of an elution buffer (50 mM HEPES pH 7.8, 100 mM NaCl, 500 mM imidazole). Protein concentration was determined using amido black (Schulz et al., 1994).

### Mass spectrometry

A total of 50 μg of the elution fraction of the *cc*FDH was prepared for mass spectrometry via filter-aided sample preparation. After tryptic digestion, the peptides were analyzed on a nanoACQUITY / SYNAPT GS-2 HDMS System, and the data were evaluated using Progenesis QI for Proteomics (Waters, Germany).

### Enzyme activity

The NADH-dependent formate production was monitored by the decrease in NADH absorbance at 340 nm, as adopted from Alissandratos et al. (2013b). In brief, 5 μM of the *cc*FDH and 0.2 mM NADH in 50 mM Bis-Tris pH 6.8 were equilibrated at 30°C to a constant absorbance at 340 nm. The reaction was then started by the addition of 100 mM sodium bicarbonate. Initial velocity within the first 2 min after induction was used to calculate kinetic parameters. The formate oxidation was measured in the presence of 0.2 mM NAD^+^ and 100 mM sodium formate under the same buffer conditions.

### Ferredoxin-dependent enzyme assay

A total of 10 μM of Fdx from *Spinacia oleracea* (soFdx) was pre-reduced by 1 mM sodium dithionite under anaerobic conditions with constant nitrogen bubbling in an Fdx buffer [50 mM Bis-Tris pH 6.8, 100 mM NaCl] for 5 min. The reaction started by the addition of 5 μM *cc*FDH and 100 mM sodium bicarbonate and stopped by heating the reaction mixture to 95°C. Protein aggregates were pelleted by centrifugation, and formate was detected in the clear supernatant.

### Formate detection

Formate was colorimetrically detected using the formate detection kit provided by Merck (Darmstadt, Germany). The assay was performed according to the manufacturer’s protocol.

### Photochronoamperometric measurements

Photochronoamperometric measurements were performed using a three-electrode system (Hartmann et al., 2018). A glassy carbon electrode, with a diameter of 4 mm, was used as the working electrode and was surrounded by a plastic tube containing the reaction medium. A Pt-wire, the counter electrode, and Ag/AgCl/3 M KCl, the reference electrode, were both placed in the reaction medium without touching the working electrode’s surface. The reaction medium included the following: 200 μL volume, 50 mM Bis-Tris pH 7.5, 30 mM NaCl, 0.03% n-Dodecyl-B-D-maltoside (*β*-DDM), 20 μM cytochrome C (Cyt c), 50 μg Chl photosystem I (PSI), 25, 50, or 100 μM *so*Fdx, 25 μM *cc*FDH, and 200 mM sodium bicarbonate (HCO_3_^−^). After the deposition of the reaction medium on the electrode, the electrode was incubated in the dark for 3 min. Subsequently, a red light of 2,300 μE at 685 nm was switched on for 30 min, followed by dark incubation for 5 min. During the measurements, the electrode was applied with a potential of 0 V vs. Ag/AgCl/ 3 M KCL. Unless otherwise specified, all post-experimental procedures were conducted in the dark or under green light.

All photochronoamperometric measurements were performed using an AUTOLAB PGSTAT12 potentiostat/galvanostat. The light intensity was controlled by adapting the applied power-on LED with the computer-based control unit PXI-1033 with the DC precision power supply module PXI-4110 (National Instruments Germany GmbH, Munich, Germany).

The proteins used for the photochronoamperometric measurements, Cytc and PSI, were purified, as mentioned previously (El-Mohsnawy et al., 2010; Medipally et al., 2023). Both PSI and Cytc were obtained from *Thermosynechococcus vestitus* BP1.

## Results and discussion

### Protein purification and enzyme assay

**Figure 1:**
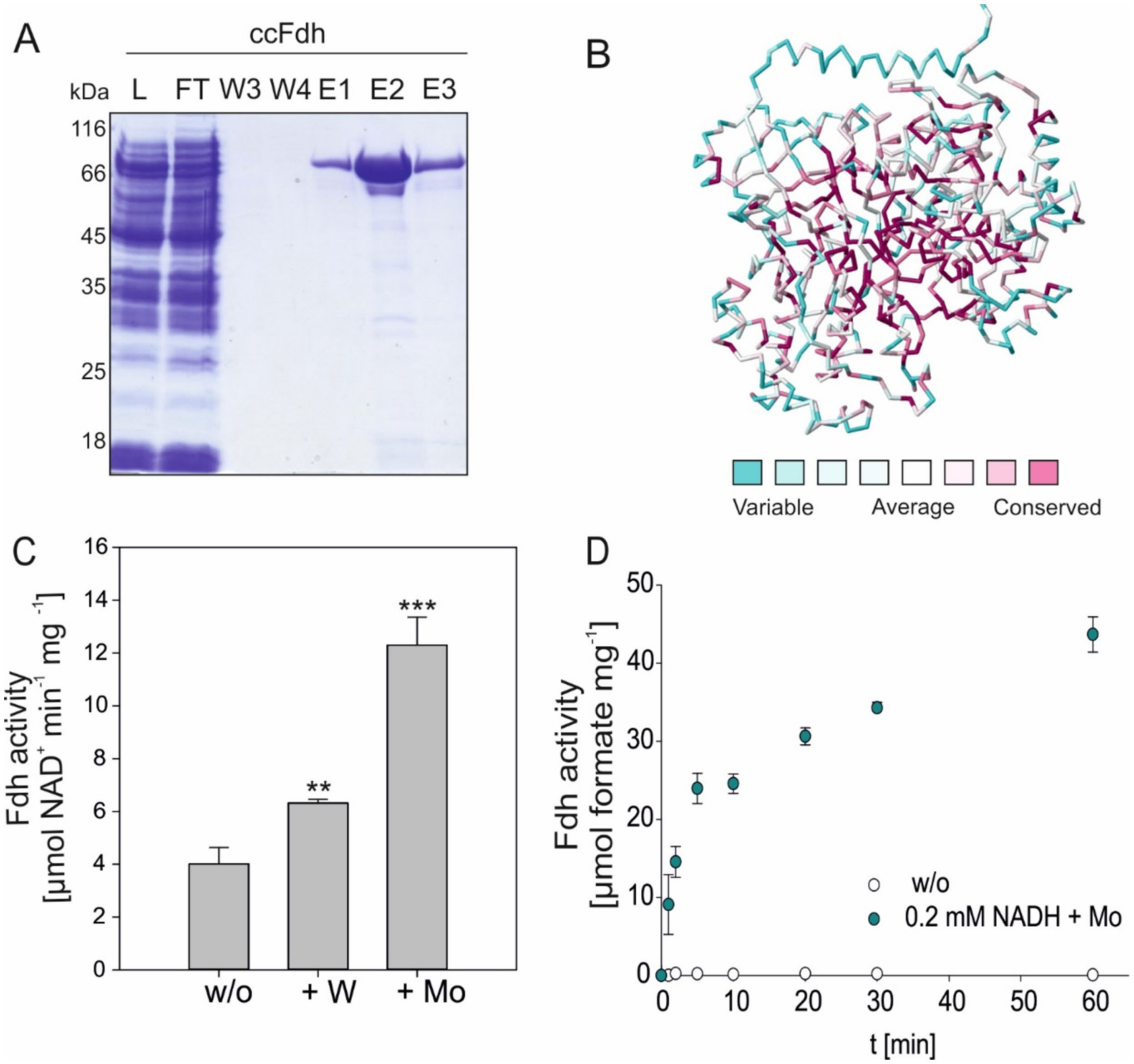
Molybdenum-dependent CO_2_ reduction by *cc*FDH. **(A)** Elution fraction after IMAC showing the degree of impurities. A representative purification is displayed. **(B)** Homology model showing the degree of conservation of the ccFDH based on the structure of ecFDH-H (PDB accession no. 1FDO). Modeling was carried out using SWISS-MODEL and the ConSurf web server. **(C)** NADH-depended CO_2_ reduction by the purified *cc*FDH obtained from the *E. coli* cultures either supplemented with 1 mM Na_2_MoO_4_, 1 mM Na_2_WO_4_, or without any addition. The FDH activity was monitored by the decrease in absorbance at 340 nm. The mean values of independent replicates (*n* = 6) are shown. ** > 0.05; *** > 0.01 **(D)** Time-dependent formate production by Mo-*cc*FDH measured at given time points using a colorimetric kit.

To assess whether the FDH from *C. carboxidivorans* (*cc*FDH) is a suitable target for expression in oxygenic phototrophs as an additional CO_2_-fixing enzyme, the biochemical features of the enzyme were investigated. For this purpose, a codon-optimized version of the *cc*FDH was cloned into the expression vector pET28a to obtain the recombinant protein with a N-terminal His_6_-tag. As reported before, the Sec codon 139Sec was exchanged for 139Cys (Alissandratos et al., 2013a) because the desired final hosts, such as cyanobacteria or plants, were not able to incorporate Sec into proteins (Zhang et al., 2006; Lopez Heras et al., 2011). The recombinant *cc*FDH was purified via IMAC as a soluble protein of approximately 80 kDa from the total protein extracts of *E. coli*. The elution fraction of the *cc*FDH revealed some proteinogenic contaminations, independent of the expression conditions (representative purification see Figure 1A). The mass spectrometry analysis revealed that these impurities did not interfere with our subsequent enzyme activity measurements because mostly chaperones and ribosomal proteins were detected (Supplementary Table S2).

For FDH activity measurements, two principal assays were applied. In the majority of the cases, the oxidation of NAD(P)H during the conversion of CO_2_ into formate was followed spectrophotometrically, which permitted measurements in the presence of different substrate and cofactor concentrations. Alternatively, the formation of the reaction product, formate, was quantified using a colorimetric formate detection kit. The second assay confirmed that the NAD(P)H oxidation in our enzyme assays specifically produced the formate. The enzyme assays were conducted under ambient oxic conditions. Furthermore, the formate oxidation activity of the *cc*FDH in general was very low and could be considered undetectable under our experimental conditions (Supplementary Figure S2).

### Molybdenum-dependent activity of *cc*FDH

Molybdenum (Mo) and tungsten (W) are the heaviest transition metals used by biological systems. They are present in the cofactors of oxidoreductases, including FDHs, due to their superior redox potential (Schwarz and Mendel, 2006). The comparison of the FDH sequences from different species revealed that the enzyme core, including the cofactor binding sites, is highly conserved (Figure 1B). According to the known crystal structures of FDH-H from *E. coli* with a verified Mo cofactor and FDH from *Desulfovibrio vulgaris* with a verified W cofactor, the Sec and neighboring His residues are thought to be involved in metal cation coordination and catalytic activity, respectively (Boyington et al., 1997; Raaijmakers et al., 2002). Alissandratos et al. (2013a) proved that the *cc*FDH harbors a W cofactor. However, the high degree of conservation in the metal cofactor-binding site made it difficult to obtain a clear structural or sequence-based prediction favoring one or other possible metals. Thus, we aimed to compare *cc*FDH-mediated CO_2_ reduction activity in the presence of either Mo or W. Furthermore, to apply the *cc*FDH to a broader host spectrum, the ability to accept Mo is of special interest.

For this purpose, the *cc*FDH was expressed in the *E. coli* cells grown in minimal M9 medium either supplemented with 1 mM Na_2_MoO_4_, 1 mM Na_2_WO_4_, or without any trace metal addition under aerobic conditions. The recombinant His-tagged protein was subsequently purified via IMAC. Using the spectrophotometric enzyme assay, the *cc*FDH expressed in the M9 medium without any supplementation revealed an activity of 4.0 ± 0.63 μmol NAD^+^ min^−1^ mg^−1^ (Figure 1C). This activity was equivalent to the initial production of approximately 4.4 μmol formate min^−1^ mg^−1^ during the first 5 min (Figure 1D). When expressed in the presence of 1 mM Na_2_WO_4_, the activity increased to 6.3 ± 0.14 μmol NAD^+^ min^−1^ mg^−1^, whereas the highest activity of 12.3 ± 1.05 μmol NAD^+^ min^−1^ mg^−1^ was measured with the 1 mM Na_2_MoO_4_ supplementation (Figure 1C). These results showed that CO_2_ reduction by the *cc*FDH did not solely rely on W, as reported previously (Alissandratos et al., 2013a), but was even enhanced in the presence of Mo. The increased catalytic rate with Mo was also observed in assays measuring the rise in the formate concentration over time. After a fast initial increase in the formate concentration, the velocity declined, but the formate was constantly produced for 1 h (Figure 1D). The detected formate concentration within the first 5 min was in good agreement with the NADH oxidation measured spectrophotometrically (Figure 1C). Hence, for all subsequent experiments, we used the *cc*FDH obtained from Mo-supplemented *E. coli* cultivations, referred to as Mo-*cc*FDH.

Next, enzyme assays with the Mo-*cc*FDH were performed in the presence of different NADH concentrations to estimate the biochemical properties of this enzyme. The obtained data were compared with the kinetic parameters for NADH oxidation in the W-cofactor-containing *cc*FDH (further referred to as W-*cc*FDH), as determined by Alissandratos et al. (2013b). The parameters for the Mo-*cc*FDH determined in our study are comparable to those determined by Alissandratos et al. (2013b) for the W-*cc*FDH (Table 1).

**Table 1:** Comparison of the enzymatic features of Mo-*cc*FDH and W-*cc*FDH.

Kinetic parameters for *cc*FDH	Mo-*cc*FDH	W-*cc*FDH^a^
K_m_ [mM]	0.06 ± 0.007	0.05
V_max_ [μmol min^−1^]	5.83 ± 0.23	5.0
k_cat_ [s^−1^]	0.02 ± 0.0008^b^	0.08
k_cat_/K_m_ [s^−1^ mM^−1^]	0.31 ± 0.084	1.60

a Data in the second column according to Alissandratos et al. (2013b).

b The smaller k_cat_ in our measurements compared to Alissandratos et al. (2013b) indicates that part of our enzyme preparation might be inactive, possibly due to insufficient metal center incorporation.

### NADPH-dependent CO_2_ reduction by *cc*FDH

**Figure 2:**
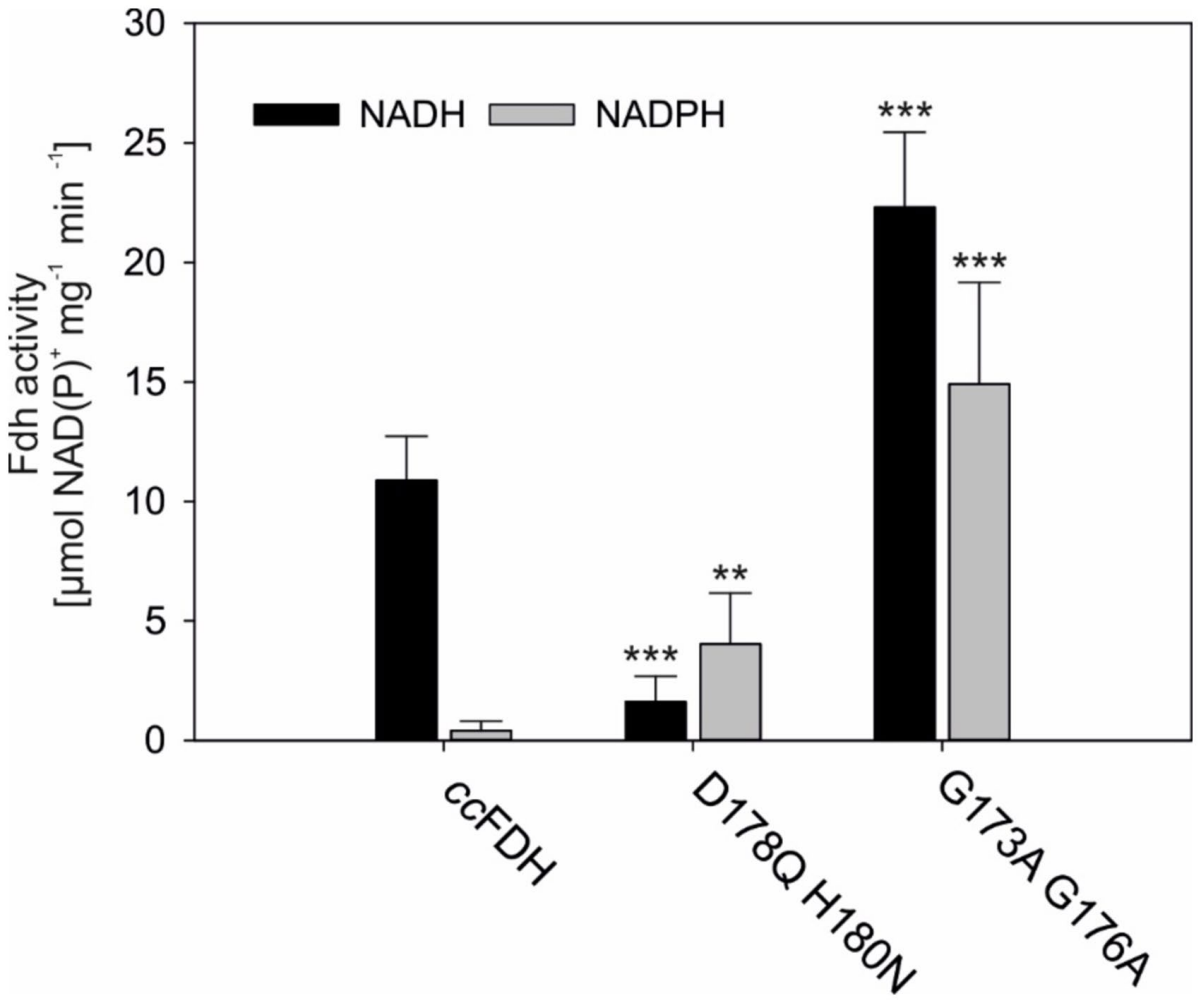
Cofactor specificity of Mo-*cc*FDH. The cofactor specificity was changed by introducing specific mutations in the nucleotide-binding site. All FDH variants were expressed in the presence of Mo. The mean values of three independent replicates and the standard deviation are shown. Significance was calculated according to the respective WT-FDH. ** > 0.05; *** > 0.01.

As mentioned above, the implementation of FDH-dependent CO_2_ fixation could support the CBB cycle and suppress photorespiration in photoautotrophs such as plants. To realize this strategy, an oxygen-tolerant FDH, such as *cc*FDH, which reduces CO_2_ to formate, needs to be expressed in photoautotrophs (Yishai et al., 2018; Song et al., 2020). However, in addition to oxygen tolerance, a well-suited FDH should be able to preferentially accept NADPH over NADH because photoautotrophs constantly produce NADPH in the photosynthetic light reactions. Hence, NADPH is the preferred reducing agent in most anabolic pathways, including CO_2_ assimilation (Park and Choi, 2017). Therefore, we aimed to change the cofactor specificity of *cc*FDH, which naturally did not show considerable activity in the presence of NADPH (Figure 2).

The nucleotide-binding site in the *cc*FDH was predicted using the Rossmann toolbox server (Kamiński et al., 2022). In *cc*FDH, it is located between amino acid residues 191 and 250 with a probability of 96.8%. The homology model revealed a typical βαβ fold within this region and a variation of the consensus nucleotide-binding sequence GxGxxxG located in a turn between β1 α1. In *cc*FDH, the consensus sequence lacks the first glycine and is composed of G_173_YNG_178_. To obtain weaker and more general cofactor binding, we introduced two glycine-to-alanine substitutions in this region, namely G173A and G178A. The variant G173A G176A showed a general increase in enzymatic activity compared to the native Mo-*cc*FDH and, as expected, was able to use NADPH with almost the same efficiency as NADH (Figure 2). In the presence of NADH, the activity of the variant G173A G176A increased two-fold compared to *cc*FDH, while its NADPH-dependent rate was 35-fold higher. Likely, the G173A G176A substitutions not only resulted in weaker and more general binding of the coenzymes but also caused faster cofactor release or exchange.

The preference of the FDH from *Pseudomonas* sp. 101 for NADH over NADPH was previously altered by exchanging D221 and H223, which are situated in close proximity to the GxGxxxG motif (Calzadiaz-Ramirez et al., 2020). Accordingly, we substituted D178 with glutamine (D178Q) and H180 with asparagine (H180N) in *cc*FDH. The amino acid substitutions in the D178Q H180N variant resulted in overall lower activity compared to the native Mo-*cc*FDH (Figure 2). However, the D178Q H180N variant showed the expected nearly two-fold higher specificity for NADPH over NADH.

### Ferredoxin-dependent CO_2_ reduction by *cc*FDH

Electron transfer between iron–sulfur clusters has been shown to be primarily dependent on the intramolecular distance between the redox centers and should ideally be placed within the boundaries of 14 Å (Page et al., 1999). Assuming that the electron-accepting FeS cluster in the *cc*FDH is located at the N-terminal domain, in close proximity to the NAD(P)-binding domain, intramolecular electron transfer mediated by ferredoxin is likely and was tested in this study.

**Figure 3:**
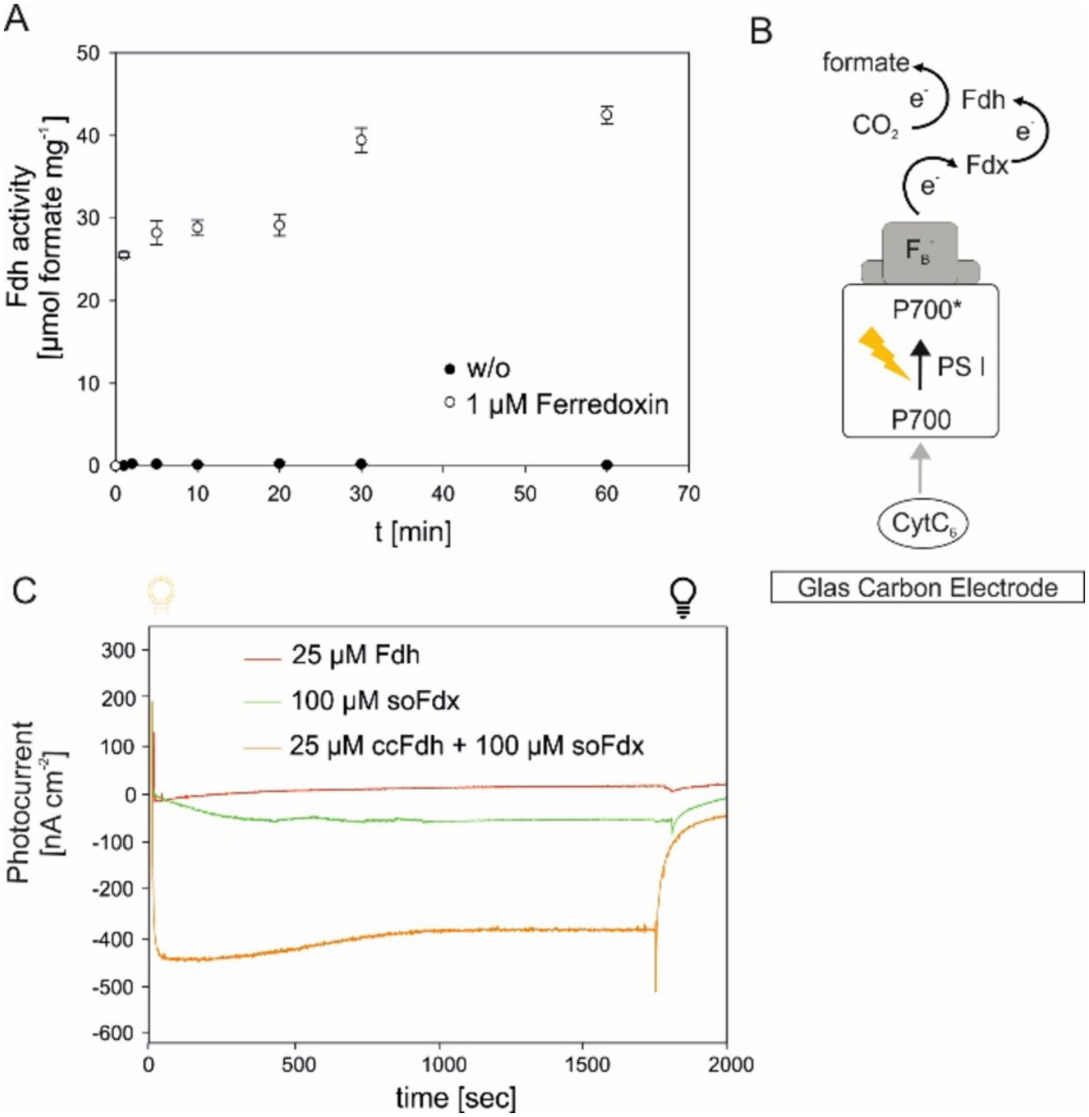
Light-dependent ferredoxin-reduction can support formate production by Mo-FDH. **(A)** Time-resolved formate production by reduced spinach ferredoxin and Mo-*cc*FDH. The mean values of three independent replicates, along with the standard deviation, are shown. **(B)** Schematic representation of photosystem I (PSI)-based photocathodes for formate formation. CytC*_6_*: cytochrome c_6_, P700*/P700: excitation of the chlorophyll (Chl) molecule of PSI upon light excitation, F_B_^−^: reduced iron–sulfur cluster. Fd, ferredoxin; FDH, formate dehydrogenase. Midpoint potential values are according to literature references (Badura et al., 2011; Kothe et al., 2013; Sokol et al., 2018). The midpoint potential of all individual components of the cascade is defined relative to the standard hydrogen electrode. **(C)** Photochronoamperometry-based reduction of ferredoxin and subsequent formate production by FDH, shown by the development of photocurrent. Each sample contained CytC, PSI, and sodium bicarbonate. The additional components are provided in the figure legend. The mean value of three independent replicates from *t* 0 s (light on, yellow bulb) to 30 min (light off, black bulb) are shown.

In the initial approach, the formate production by the Mo-*cc*FDH was measured in the presence of 10 μM of ferredoxin from *Spinacia oleracea* (*so*Fdx), reduced by sodium dithionite. Under our assay conditions, the enzyme was able to produce up to 42 μg of the formate within 1 h (Figure 3A). The initial reaction velocity, approximately 25.5 μmol min^−1^, exceeded that of the NADH-dependent formate production by 2.8-fold. However, the absolute formate concentration after 1 hour remained nearly the same. Most likely, the reduced *so*Fdx was not stable for extended periods under our test conditions because oxygen could not be completely omitted from the assays, leading to *so*Fdx oxidation.

Next, we aimed to investigate whether it is possible to couple ferredoxin-mediated electron transport from photosynthesis with Mo-*cc*FDH-mediated CO_2_ reduction. This was tested by the artificial reconstitution of the cathodic part of the photosynthetic electron transport chain, using a carbon electrode, CytC, photosystem I (PSI), *so*Fdx, *cc*FDH, and CO_2_. The difference in the mid-point potentials between these components created a paradigm for electron transfer, as shown in Figure 3B. In this cascade, the initial electron donor was a carbon electrode with a mid-point potential of 0 V versus Ag/AgCl/ 3 M KCl and the final electron acceptor was CO_2_. The electron transfer within this cascade was monitored by changes in the photocurrent density. Upon light excitation, the photo-energized PSI initiated the rapid uptake of the electrons from the electrode via Cytc, which was visible by the formation of a negative current (Figure 3C). Then, the PSI-driven energized electrons were transferred via the *so*Fdx to Mo-*cc*FDH, which further reduced CO_2_ to formate. When only the soFdx was added to this cascade (*so*Fdx, green in Figure 3C), we observed a low photocurrent density of −29.15 nA cm^−2^ after 5 min of illumination, indicating a compromise in the electron consumption of any of the individual components in the cascade. When only the Mo-*cc*FDH was added, the current showed no significant decrease, with a value of −13.48 nA cm^−2^, after 5 min of illumination (*cc*FDH, blue in Figure 3C). However, when all components of the cascade were added, we observed the highest photocurrent density of −460.44 nA cm^−2^ after 5 min illumination, indicating electron consumption by the *so*Fdx-dependent *cc*FDH. Here, the rate-limiting component was the *so*Fdx (compared to Supplementary Figure S3), most likely due to the inefficient reduction of the plant Fdx by cyanobacterial PSI. These experiments clearly showed that the native *cc*FDH can convert CO_2_ into formate using a reduced Fdx, which is available in high amounts during the light period in photoautotrophs. However, the Fdx-dependent coupling of CO_2_ reduction via the *cc*FDH may have regulatory effects on the photosynthetic electron flow *in vivo*, which needs to be analyzed in future experiments once the enzyme is successfully expressed in a photoautotrophic cell.

## Conclusion

The FDH from *Clostridium carboxidivorans* investigated in this study has the capability to reduce CO_2_ under aerobic conditions with high efficiency, while the reverse reaction is virtually absent. Moreover, in addition to W, it can also accept Mo as the transition metal in its pyranopterin cofactor, making it suitable for use in any organism that contains this cofactor family, such as most phototrophs, which use the Mo-cofactor in their nitrate reductase (Schwarz and Mendel, 2006). The cofactor preference of the *cc*FDH can be altered by a few amino acid substitutions in the Rossmann fold, enabling it to efficiently use NADPH in addition to NADH, which is produced in high amounts during photosynthetic light reactions. Finally, the native *cc*FDH can also directly use Fdx as a reducing agent, which is generated by the photosynthetic light reaction at PSI. These data collectively suggest that *cc*FDH and, particularly, slightly optimized variants can be regarded as suitable enzymes to couple formate production to photosynthesis in photoautotroph organisms, potentially supporting CO_2_ assimilation via the CBB cycle and minimizing CO_2_ losses due to photorespiration.

## Data Availability

The original contributions presented in the study are included in the article/Supplementary material, further inquiries can be directed to the corresponding authors.
