# Supplementary material for: Characterization of the oxygen-tolerant formate dehydrogenase from Clostridium carboxidivorans
Source: Front Microbiol. 2025 Jan 13;15:1527626. doi: 10.3389/fmicb.2024.1527626 (PMC11770034; doi:10.3389/fmicb.2024.1527626)
Supplement: Supplementary file 1 [file Data_Sheet_1.pdf]

Supplementary Material for the manuscript

Characterization of an oxygen-tolerant formate dehydrogenase from *Clostridium carboxidivorans*

Eva-Maria Brouwer[1]*, Hitesh K. R. Medipally[2,3], Saskia Schwab[1], Shanshan Song[1], Marc M. Nowaczyk[2.4,5], and Martin Hagemann[1,5]*

[1] University of Rostock, Institute of Biosciences, Plant Physiology, A.-Einstein-Str. 3, D-18059 Rostock, Germany; [2] Plant Biochemistry, Faculty of Biology and Biotechnology, Ruhr-University Bochum, D-44780 Bochum, Germany; [3] School of Engineering Sciences in Chemistry, Biotechnology and Health, Science for Life Laboratory, KTH—Royal Institute of Technology, Stockholm, Sweden; [4] University of Rostock, Institute of Biosciences, Biochemistry, A.-Einstein-Str. 3, D-18059 Rostock, Germany; [5] Interdisciplinary Faculty, Department Life, Light and Matter, University of Rostock, Rostock, Germany

*corresponding authors: Eva-Maria Brouwer; University of Rostock, Institute of Biosciences, Dept. Plant Physiology, A.-Einstein-Str. 3, D-18059 Rostock, Germany, Tel. +49(0)3814986115, Fax. +49(0)3814986112, Email: eva-maria.brouwer@uni-rostock.de. Martin Hagemann; University of Rostock, Institute of Biosciences, Dept. Plant Physiology, A.-Einstein-Str. 3, D-18059 Rostock, Germany, Tel. +49(0)3814986110, Fax. +49(0)3814986112, Email: martin.hagemann@uni-rostock.de.

Supplementary Figures S1-S3

```
ATGGGCAGCAGCCATCATCATCATCATCACAGCAGCGGCCTGGTGCCGCGCGGCAGCCAT
ATGGCTAGCTGGAGCCACCCGCAGTTCGAAAAAATCGAAGGGCGCCGAGACCGCGGTCCC
GAATTCGAAAACAAAGTGTTGACCGTGTGTCCCTACTGTGGCGCCGGCTGTAACTTGTAC
TTGGTGGTGGAAGATGGCAAAATTGTGCGCGCCGAACCCGCCAACGGCCGCACCAACGAA
GGCAACTTGTGTTTGAAAGGCCACTACGGCTGGGATTTTTTGAACGATCCCAAAATTTTG
ACCTCCCGCTTGAAAAAACCCATGATTCGCAAAAACGGCCAATTGGAAGAAGTGTCCTGG
GAAGAAGCCATTGGCTTTACCGCCTCCAAATTGAAAGAAATTAAAGAAAAATACGGCCCC
GATTCCATTATGGGCACCGGCTGTGCCCGCGGCTCCGGCAACGAAGCCAACTACATTATG
CAAAAATTTATGCGCGCCGTGATTGGCACCAACAACGTGGATCACTGTGCCCGCGTGTGT
CACGCCCCCTCCGTGGCCGGCTTGGCCTACGTGTTGGGCAACGGCGCCATGTCCAACGGC
ATTCACGAAATTGATGATTGTGATTTGGTGTTTATTTTTGGCTACAACGGCGCCGATTCC
                                       G192    G197  D199
CACCCCATTGTGGCCCGCCGCATTGTGAACGCCAAACGCAAAGGCGCCAAAATTGTGGTG
H201
ACCGATCCCCGCATTACCGAATCCGCCCGCATTGCCGATTTGTGGTTGCCCATTAAAAAC
GGCACCAACATGATTTTGGTGAACGCCTTTGCCAACGTGTTGATTAACGAAGGCTTGTAC
AACAAACAATACGTGGAAGAACACACCGTGGGCTTTGAAGAATACAAAGCCTTGGTGGAA
AAATACACCCCCGAATACGCCGAAAAAATGACCGGCGTGCCCGCCGAAGATATTCGCAAA
TCCATGCGCATGTACTCCAAAGCCAAAAACGCCATGATTTTGTACGGCATGGGCGTGTGT
CAATTTGGCCAAGCCGTGGATGTGGTGAAAGGCTTGGCCTCCTTGGCCTTGTTGACCGGC
AACTTTGGCCGCCCCAACGTGGGCATTGGCCCCGTGCGCGGCCAAAACAACGTGCAAGGC
GCCTGTGATATGGGCGCCTTGCCCAACGTGTACCCCGGCTACCAATCCGTGACCAACGAT
GCCATTCGCGAAAAATTTGAAAACGCCTGGGGCGTGAAATTGCCCAACAAAGTGGGCTAC
CACTTGACCGAAGTGCCCCACTTGGTGTTGAAAGAAGATAAAATTAAAGCCTACTACATT
ATGGGCGAAGATCCCGTGCAATCCGATCCCGATGCCGCCGAAGTGCGCGAAGCCTTGGAT
AAATTGGAATTGGTGATTGTGCAAGATATTTTTATGAACAAAACCGCCTTGCACGCCGAT
GTGATTTTGCCCGCCACCTCCTGGGGCGAACACGAAGGCGTGTACTCCTCCGCCGATCGC
GGCTTTCAACGCTTTCGCAAAGCCATTGAACCCACCGGCGATGTGAAACCCGATTGGCAA
ATTATTTCCGAAATTGCCAAAGCCATGGGCTACGATATGAACTACAAAAACACCAAAGAA
ATTTGGGATGAATTGCGCAACTTGTGTCCCAACTTTAAAGGCGCCTCCTACGAACGCTTG
GAAGAATTGGGCGGCATTCAATGGCCCTGTCCCTCCGAAGATCACCCCGGCACCTCCTAC
TTGTACAAAGGCAACAAATTTAACACCCCCTCCGGCAAAGCCAACTTGTTTGCCGCCGAA
TGGCGCGCCCCCATGGAATCCACCGATAAAGAATACCCCTTGGTGTTGTCCACCGTGCGC
GAAGTGGGCCACTACTCCGTGCGCACCATGACCGGCAACTGTCGCGCCTTGCAACAATTG
GCCGATGAACCCGGCTACGTGCAAATTAACCCCGAAGATGCCAAAAACTTGAACATTTTG
GATCAAGAATTTGTGCGCATTTCCTCCCGCCGCGGCTCCGTGGTGGCCAAAGCCTTGGTG
ACCGATCGCGTGAACAAAGGCGCCGTGTACATGACCTACCAATGGTGGGTGGGCGCCTGT
AACGAATTGACCTTGAACAACTTGGATCCCATTTCCAAAACCCCCGAATACAAATACTGT
GCCGTGAAAGTGGAAAACATTAAAGATCAAAAAGCCGCCGAACAATACGTGCAAGATGAA
TACACCAAAATTCGCAAAAAAATGAACATTAACTTGGAATGTTGTAAATAA
```

Figure S1: Sequence of the codon-optimized *cc*Fdh. Substituted codons are highlighted in blue.

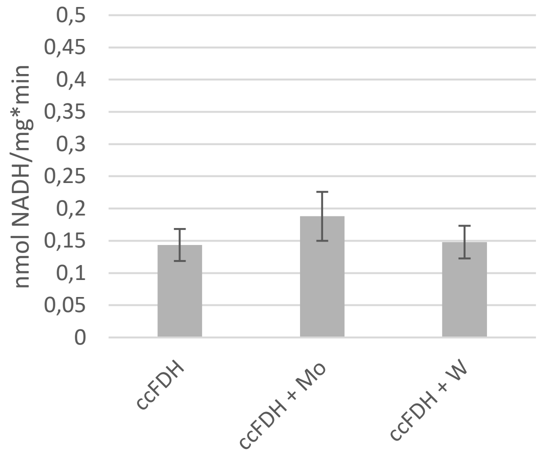

Figure S2: Formate reduction activity of *cc*Fdh under our experimental conditions. Experiments were performed in the presence of 100 mM sodium format and 0.2 mM NAD+.

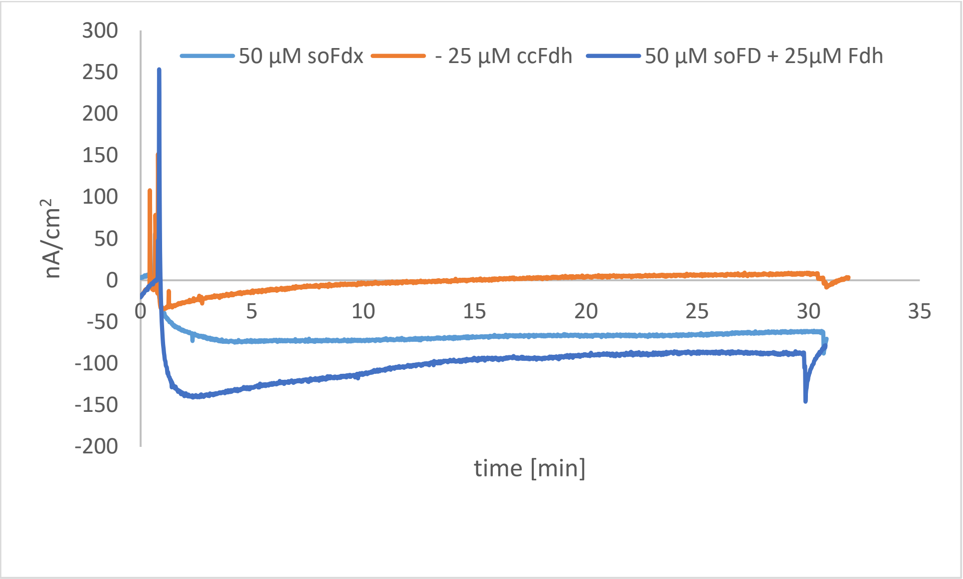

Figure S3: Photochronoamperometry based reduction of ferredoxin and subsequent formate production by Fdh shown by the development of photocurrent. Each sample contained CytC, PSI and sodium bicarbonate. The additional components are given in the figure legend. Shown are the mean value of three independent replicates from t 0 sec (light on,) to 30 min (light off).

Supplementary Tables S1-S2

Table S1: Primers for side directed mutagenesis. Silent restriction sides were introduced for evaluation and indicated in the primer name.

Name	Sequence
**D199Q-H201N_MunI**	GGCTACAACGGCGCCcaaTCCaacCCaATTGTGGCCCGCCGC
**G192A-G197A_SacII**	GTGATTTGGTGTTTATTTTTgccTACAACgccGCgGATTCCCACCCCATTGTGG

Table S2: Mass spectrometry of the elution fraction of *cc*Fdh after IMAC purification.

Accession	Peptide count	Unique peptides	Confidence score	Mass	Description
X00000	62	62	887,0	83267	Clostridium carboxidivorans codon optimiert SV=1
P0A6Y8	50	50	626,3	69172	Chaperone protein DnaK OS=Escherichia coli (strain K12) GN=dnaK PE=1 SV=2
P0A6F5	40	40	500,8	57500	60 kDa chaperonin OS=Escherichia coli (strain K12) GN=groL PE=1 SV=2
P17169	30	30	320,9	67123	Glutamine--fructose-6-phosphate aminotransferase [isomerizing] OS=Escherichia coli (strain K12) GN=glmS PE=1 SV=4
P77398	29	29	378,1	74916	Bifunctional polymyxin resistance protein ArnA OS=Escherichia coli (strain K12) GN=arnA PE=1 SV=1
P0AG30	24	24	281,7	47061	Transcription termination factor Rho OS=Escherichia coli (strain K12) GN=rho PE=1 SV=1
P0ACJ8	21	21	317,3	23812	cAMP-activated global transcriptional regulator CRP OS=Escherichia coli (strain K12) GN=crp PE=1 SV=1
P0AFG8	17	17	114,6	100011	Pyruvate dehydrogenase E1 component OS=Escherichia coli (strain K12) GN=aceE PE=1 SV=2
P0AG67	16	16	139,3	61272	30S ribosomal protein S1 OS=Escherichia coli (strain K12) GN=rpsA PE=1 SV=1
P0A850	15	15	117,4	48193	Trigger factor OS=Escherichia coli (strain K12) GN=tig PE=1 SV=1
P0A9P0	13	13	106,0	50974	Dihydrolipoyl dehydrogenase OS=Escherichia coli (strain K12) GN=lpdA PE=1 SV=2
P0A9J8	12	12	95,2	43340	P-protein OS=Escherichia coli (strain K12) GN=pheA PE=1 SV=1
P0AFG3	11	11	72,8	105632	2-oxoglutarate dehydrogenase E1 component OS=Escherichia coli (strain K12) GN=sucA PE=1 SV=1
P39451	11	11	108,5	35893	Alcohol dehydrogenase_ propanol-preferring OS=Escherichia coli (strain K12) GN=adhP PE=1 SV=1
P0A7R1	10	10	90,7	15769	50S ribosomal protein L9 OS=Escherichia coli (strain K12) GN=rplI PE=1 SV=1
P0A7V8	10	10	99,0	23526	30S ribosomal protein S4 OS=Escherichia coli (strain K12) GN=rpsD PE=1 SV=2
P0A991	10	10	94,4	38337	Fructose-bisphosphate aldolase class 1 OS=Escherichia coli (strain K12) GN=fbaB PE=1 SV=2
P60422	10	10	83,2	29975	50S ribosomal protein L2 OS=Escherichia coli (strain K12) GN=rplB PE=1 SV=2
P60438	10	10	115,9	22244	50S ribosomal protein L3 OS=Escherichia coli (strain K12) GN=rplC PE=1 SV=1
P06959	9	9	60,3	66153	Dihydrolipoyllysine-residue acetyltransferase component of pyruvate dehydrogenase complex OS=Escherichia coli (strain K12) GN=aceF PE=1 SV=3
P31658	9	9	96,4	31305	Protein deglycase 1 OS=Escherichia coli (strain K12) GN=hchA PE=1 SV=3
P62399	9	9	74,1	20359	50S ribosomal protein L5 OS=Escherichia coli (strain K12) GN=rplE PE=1 SV=2

P0A7L0	8	8	62,6	24730	50S ribosomal protein L1 OS=Escherichia coli (strain K12) GN=rplA PE=1 SV=2
P0A7V0	8	8	65,8	26801	30S ribosomal protein S2 OS=Escherichia coli (strain K12) GN=rpsB PE=1 SV=2
P0A9K9	8	8	83,1	21195	FKBP-type peptidyl-prolyl cis-trans isomerase SlyD OS=Escherichia coli (strain K12) GN=slyD PE=1 SV=1
P0A9S5	8	8	61,5	39111	Glycerol dehydrogenase OS=Escherichia coli (strain K12) GN=gldA PE=1 SV=1
P0A858	8	8	93,8	27143	Triosephosphate isomerase OS=Escherichia coli (strain K12) GN=tpiA PE=1 SV=1
P0AFG6	8	8	92,7	44011	Dihydrolipoyllysine-residue succinyltransferase component of 2-oxoglutarate dehydrogenase complex OS=Escherichia coli (strain K12) GN=sucB PE=1 SV=2
P0AG55	8	8	74,5	18961	50S ribosomal protein L6 OS=Escherichia coli (strain K12) GN=rplF PE=1 SV=2
P02931	8	8	77,6	39333	Outer membrane protein F OS=Escherichia coli (strain K12) GN=ompF PE=1 SV=1
P0A7J3	7	7	54,1	17769	50S ribosomal protein L10 OS=Escherichia coli (strain K12) GN=rplJ PE=1 SV=2
P0A7S9	7	7	78,5	13156	30S ribosomal protein S13 OS=Escherichia coli (strain K12) GN=rpsM PE=1 SV=2
P0A7V3	7	7	60,6	25983	30S ribosomal protein S3 OS=Escherichia coli (strain K12) GN=rpsC PE=1 SV=2
P0A7X3	7	7	52,5	14856	30S ribosomal protein S9 OS=Escherichia coli (strain K12) GN=rpsI PE=1 SV=2
P0AA10	7	7	71,1	16019	50S ribosomal protein L13 OS=Escherichia coli (strain K12) GN=rplM PE=1 SV=1
P0ACR4	7	7	63,5	32895	Uncharacterized HTH-type transcriptional regulator YeiE OS=Escherichia coli (strain K12) GN=yeiE PE=3 SV=1
P02359	7	7	79,7	20019	30S ribosomal protein S7 OS=Escherichia coli (strain K12) GN=rpsG PE=1 SV=3
P60723	7	7	67,0	22087	50S ribosomal protein L4 OS=Escherichia coli (strain K12) GN=rplD PE=1 SV=1
P61175	7	7	72,9	12226	50S ribosomal protein L22 OS=Escherichia coli (strain K12) GN=rplV PE=1 SV=1
P68919	7	7	63,2	10693	50S ribosomal protein L25 OS=Escherichia coli (strain K12) GN=rplY PE=1 SV=1
B8LFD6;P00722	6	6	38,6	117395	Beta-galactosidase OS=Escherichia coli (strain K12) PE=3 SV=1
P0A7W1	6	6	59,5	17603	30S ribosomal protein S5 OS=Escherichia coli (strain K12) GN=rpsE PE=1 SV=2
P0A9A6	6	6	41,3	40324	Cell division protein FtsZ OS=Escherichia coli (strain K12) GN=ftsZ PE=1 SV=1
P0A910	6	6	38,8	37315	Outer membrane protein A OS=Escherichia coli (strain K12) GN=ompA PE=1 SV=1
P0ADY3	6	6	74,1	13655	50S ribosomal protein L14 OS=Escherichia coli (strain K12) GN=rplN PE=1 SV=1
P0CE47;P0CE48	6	6	39,8	43455	Elongation factor Tu 1 OS=Escherichia coli (strain K12) GN=tufA PE=1 SV=1
P25552	6	6	39,0	55327	Guanosine-5'-triphosphate_3'-diphosphate pyrophosphatase OS=Escherichia coli (strain K12) GN=gppA PE=1 SV=3
P64588	6	6	70,0	23858	Transcriptional regulator YqjI OS=Escherichia coli (strain K12) GN=yqjI PE=1 SV=1
P0A6Z3	5	5	29,4	71423	Chaperone protein HtpG OS=Escherichia coli (strain K12) GN=htpG PE=1 SV=1

P0A7J7	5	5	49,0	14932	50S ribosomal protein L11 OS=Escherichia coli (strain K12) GN=rplK PE=1 SV=2
P0A7R5	5	5	58,2	11736	30S ribosomal protein S10 OS=Escherichia coli (strain K12) GN=rpsJ PE=1 SV=1
P0A7U7	5	5	38,7	9684	30S ribosomal protein S20 OS=Escherichia coli (strain K12) GN=rpsT PE=1 SV=2
P0A9A9	5	5	50,2	17023	Ferric uptake regulation protein OS=Escherichia coli (strain K12) GN=fur PE=1 SV=1
P0AA43	5	5	53,1	25979	Ribosomal small subunit pseudouridine synthase A OS=Escherichia coli (strain K12) GN=rsuA PE=1 SV=1
P0AG44	5	5	38,7	14422	50S ribosomal protein L17 OS=Escherichia coli (strain K12) GN=rplQ PE=1 SV=1
P02358	5	5	39,2	15704	30S ribosomal protein S6 OS=Escherichia coli (strain K12) GN=rpsF PE=1 SV=1
P02413	5	5	38,2	14980	50S ribosomal protein L15 OS=Escherichia coli (strain K12) GN=rplO PE=1 SV=1
P09373	5	5	30,9	85643	Formate acetyltransferase 1 OS=Escherichia coli (strain K12) GN=pflB PE=1 SV=2
P27306	5	5	39,3	52017	Soluble pyridine nucleotide transhydrogenase OS=Escherichia coli (strain K12) GN=sthA PE=1 SV=5
P77567	5	5	43,7	32503	N-hydroxyarylamine O-acetyltransferase OS=Escherichia coli (strain K12) GN=nhoA PE=1 SV=1
P0A6F9	4	4	40,0	10387	10 kDa chaperonin OS=Escherichia coli (strain K12) GN=groS PE=1 SV=1
P0A6X3	4	4	38,3	11166	RNA-binding protein Hfq OS=Escherichia coli (strain K12) GN=hfq PE=1 SV=2
P0A7K6	4	4	39,0	13133	50S ribosomal protein L19 OS=Escherichia coli (strain K12) GN=rplS PE=1 SV=2
P0A7T7	4	4	41,1	9043	30S ribosomal protein S18 OS=Escherichia coli (strain K12) GN=rpsR PE=1 SV=2
P0A8P8	4	4	27,2	34246	Tyrosine recombinase XerD OS=Escherichia coli (strain K12) GN=xerD PE=1 SV=1
P0ADY7	4	4	30,1	15281	50S ribosomal protein L16 OS=Escherichia coli (strain K12) GN=rplP PE=1 SV=1
P0ADZ4	4	4	36,3	10269	30S ribosomal protein S15 OS=Escherichia coli (strain K12) GN=rpsO PE=1 SV=2
P0C018	4	4	45,1	12770	50S ribosomal protein L18 OS=Escherichia coli (strain K12) GN=rplR PE=1 SV=1
P05055	4	4	23,8	77158	Polyribonucleotide nucleotidyltransferase OS=Escherichia coli (strain K12) GN=pnp PE=1 SV=3
P32664	4	4	34,5	30031	NADH pyrophosphatase OS=Escherichia coli (strain K12) GN=nudC PE=1 SV=2
P68679	4	4	26,6	8557	30S ribosomal protein S21 OS=Escherichia coli (strain K12) GN=rpsU PE=1 SV=2
P0A6Q6	3	3	23,1	17147	3-hydroxyacyl-[acyl-carrier-protein] dehydratase FabZ OS=Escherichia coli (strain K12) GN=fabZ PE=1 SV=1
P0A6V8	3	3	18,8	35065	Glucokinase OS=Escherichia coli (strain K12) GN=glk PE=1 SV=1
P0A6Y5	3	3	21,5	32877	33 kDa chaperonin OS=Escherichia coli (strain K12) GN=hslO PE=1 SV=1
P0A7K2	3	3	24,1	12295	50S ribosomal protein L7/L12 OS=Escherichia coli (strain K12) GN=rplL PE=1 SV=2

P0A7L8	3	3	20,4	9182	50S ribosomal protein L27 OS=Escherichia coli (strain K12) GN=rpmA PE=1 SV=2
P0A7M6	3	3	30,8	7273	50S ribosomal protein L29 OS=Escherichia coli (strain K12) GN=rpmC PE=1 SV=1
P0A7M9	3	3	32,2	8099	50S ribosomal protein L31 OS=Escherichia coli (strain K12) GN=rpmE PE=1 SV=1
P0A7R9	3	3	28,3	13959	30S ribosomal protein S11 OS=Escherichia coli (strain K12) GN=rpsK PE=1 SV=2
P0A7S3	3	3	31,2	13965	30S ribosomal protein S12 OS=Escherichia coli (strain K12) GN=rpsL PE=1 SV=2
P0A7T3	3	3	19,7	9191	30S ribosomal protein S16 OS=Escherichia coli (strain K12) GN=rpsP PE=1 SV=1
P0A7Z4	3	3	20,6	36740	DNA-directed RNA polymerase subunit alpha OS=Escherichia coli (strain K12) GN=rpoA PE=1 SV=1
P0ACC3	3	3	22,1	12272	Iron-sulfur cluster insertion protein ErpA OS=Escherichia coli (strain K12) GN=erpA PE=1 SV=1
P0ACF0	3	3	20,8	9535	DNA-binding protein HU-alpha OS=Escherichia coli (strain K12) GN=hupA PE=1 SV=1
P0ADZ0	3	3	22,5	11199	50S ribosomal protein L23 OS=Escherichia coli (strain K12) GN=rplW PE=1 SV=1
P0AEI1	3	3	17,7	54005	tRNA-2-methylthio-N(6)-dimethylallyladenosine synthase OS=Escherichia coli (strain K12) GN=miaB PE=1 SV=1
P0AG48	3	3	21,1	11564	50S ribosomal protein L21 OS=Escherichia coli (strain K12) GN=rplU PE=1 SV=1
P23908	3	3	24,1	42804	Acetylornithine deacetylase OS=Escherichia coli (strain K12) GN=argE PE=1 SV=2
P24252	3	3	20,2	20211	Uncharacterized protein YbgA OS=Escherichia coli (strain K12) GN=ybgA PE=4 SV=1
P60624	3	3	21,5	11316	50S ribosomal protein L24 OS=Escherichia coli (strain K12) GN=rplX PE=1 SV=2
P69776	3	3	33,0	8381	Major outer membrane lipoprotein Lpp OS=Escherichia coli (strain K12) GN=lpp PE=1 SV=1
P76270	3	3	18,9	18293	Free methionine-R-sulfoxide reductase OS=Escherichia coli (strain K12) GN=msrC PE=1 SV=2
P77552	3	3	19,1	43161	Uncharacterized protein YdhQ OS=Escherichia coli (strain K12) GN=ydhQ PE=4 SV=1
C3UPD1;P0A832	2	2	13,6	17333	SsrA-binding protein (Fragment) OS=Escherichia coli (strain K12) GN=smpB PE=3 SV=1
P0A6T5	2	2	11,9	24945	GTP cyclohydrolase 1 OS=Escherichia coli (strain K12) GN=folE PE=1 SV=2
P0A7B3	2	2	11,2	32909	NAD kinase OS=Escherichia coli (strain K12) GN=nadK PE=1 SV=1
P0A7G6	2	2	12,9	38145	Protein RecA OS=Escherichia coli (strain K12) GN=recA PE=1 SV=2
P0A7L3	2	2	15,8	13497	50S ribosomal protein L20 OS=Escherichia coli (strain K12) GN=rplT PE=1 SV=2
P0AEU7	2	2	13,3	17688	Chaperone protein Skp OS=Escherichia coli (strain K12) GN=skp PE=1 SV=1
P0AFZ3	2	2	15,9	18262	Stringent starvation protein B OS=Escherichia coli (strain K12) GN=sspB PE=1 SV=1

P0AG51	2	2	21,6	6542	50S ribosomal protein L30 OS=Escherichia coli (strain K12) GN=rpmD PE=1 SV=2
P0AG63	2	2	30,3	9819	30S ribosomal protein S17 OS=Escherichia coli (strain K12) GN=rpsQ PE=1 SV=2
P0C0V0	2	2	12,5	49468	Periplasmic serine endoprotease DegP OS=Escherichia coli (strain K12) GN=degP PE=1 SV=1
P36999	2	2	18,2	30875	23S rRNA (guanine(745)-N(1))-methyltransferase OS=Escherichia coli (strain K12) GN=rlmA PE=1 SV=1
P63020	2	2	13,3	21226	Fe/S biogenesis protein NfuA OS=Escherichia coli (strain K12) GN=nfuA PE=1 SV=1
P63417	2	2	13,1	18591	Uncharacterized N-acetyltransferase YhbS OS=Escherichia coli (strain K12) GN=yhbS PE=3 SV=1
P0A7W7	1	1	6,2	14184	30S ribosomal protein S8 OS=Escherichia coli (strain K12) GN=rpsH PE=1 SV=2
P0A962	1	1	5,8	37184	L-asparaginase 1 OS=Escherichia coli (strain K12) GN=ansA PE=1 SV=1
P0ACP1	1	1	6,0	38171	Catabolite repressor/activator OS=Escherichia coli (strain K12) GN=cra PE=1 SV=1
P0C8J8	1	1	6,1	47565	D-tagatose-1_6-bisphosphate aldolase subunit GatZ OS=Escherichia coli (strain K12) GN=gatZ PE=1 SV=1
P21179	1	1	6,2	84277	Catalase HPII OS=Escherichia coli (strain K12) GN=katE PE=1 SV=1
P76344	1	1	6,1	24876	Metal-binding protein ZinT OS=Escherichia coli (strain K12) GN=zinT PE=1 SV=1
